# Evaluation of a Risk Stratification Model Using Preoperative and Intraoperative Data for Major Morbidity or Mortality After Cardiac Surgical Treatment

**DOI:** 10.1001/jamanetworkopen.2020.28361

**Published:** 2020-12-07

**Authors:** Thomas J. S. Durant, Raymond A. Jean, Chenxi Huang, Andreas Coppi, Wade L. Schulz, Arnar Geirsson, Harlan M. Krumholz

**Affiliations:** 1 Center for Outcomes Research and Evaluation, Yale New Haven Hospital, New Haven, Connecticut; 2 Department of Laboratory Medicine, Yale University School of Medicine, New Haven, Connecticut; 3 Department of Surgery, Yale University School of Medicine, New Haven, Connecticut; 4 Division of Cardiac Surgery, Department of Surgery, Yale University School of Medicine, New Haven, Connecticut; 5 Section of Cardiovascular Medicine, Department of Internal Medicine, Yale School of Medicine, New Haven, Connecticut; 6 Department of Health Policy and Management, Yale School of Public Health, New Haven, Connecticut

## Abstract

This cohort study evaluates performance of risk stratification models using preoperative, intraoperative, or combined data to assess composite risk after cardiac surgical treatment.

## Introduction

Postoperative risk–stratification models for major surgical procedures are the standard of care for operative care planning. Risk models, such as the Society of Thoracic Surgeons (STS) Adult Cardiac Surgery Database (ACSD) coronary artery bypass risk calculator, rely on preoperative clinical data to stratify patients by risk for complications.^1,2^ While operative strategy and intraoperative events may influence outcomes, the degree to which intraoperative data could be used to improve postoperative risk stratification is not well characterized.^3^ Accordingly, we used information from a single-center STS-ACSD registry to investigate whether use of intraoperative variables was associated with improved accuracy in postoperative risk stratification.

## Methods

The institutional review board of Yale University approved this cohort study and waived the informed consent requirement for the use of retrospective data from the STS registry given the retrospective nature of the study and the deidentified nature of the analysis. This study followed the Strengthening the Reporting of Observational Studies in Epidemiology (STROBE) reporting guideline. Data were extracted from our local STS ACSD database, version 2.81, from 2011 to 2017 for patients who received coronary artery bypass with or without a concurrent valve procedure.

We adapted initial preoperative variable selection, adverse events, and data imputation from O’Brien et al^1^ and Shahian et al.^2^ Intraoperative candidate variables are defined in the ACSD version 2.81 data collection form. From the candidate variables, we used least absolute shrinkage and selection operator (LASSO) regularization^4^ for variable selection to develop 3 separate logistic regression models that made use of preoperative variables, intraoperative variables, or a combination of preoperative and intraoperative variables. We evaluated the ability of these 3 models to stratify patients by the probability that they would experience a composite of major morbidity or mortality events (eg, operative mortality or reoperation), as previously described.^2^ Performance was assessed using the area under the receiver operator characteristic curve (AUROC) and F1 score (ie, the harmonic mean of positive predictive value and sensitivity) across 100 random splits of the data into training and validation sets. Differences in mean performance metrics between models were assessed using 2-sided unpaired independent *t* tests with Bonferroni corrections to account for multiple comparisons, and significance was set at P = .016. Data preprocessing and statistical analysis were performed with Python version 2.7 (Python Software Foundation) and the scikit-learn software package version 0.19.1.^5^ Data analysis was performed October 9, 2018, to May 20, 2019.

## Results

Of 2905 individuals in the analysis (mean [SD] age, 67.8 [10.6] years; 2193 [75.4%] men), 465 patients experienced an adverse event, for a composite event rate of 15.9%. Of preoperative and intraoperative candidate variables, LASSO regularization identified important variables (ie, those with higher coefficients weighted more heavily by the model) for each of the 3 models (Table).^4^ The magnitude of importance was determined by the distance of the variable coefficient from zero. The intraoperative-only model, compared with the baseline preoperative model, had significantly lower mean (SD) AUROC (0.74 [0.02] vs 0.75 [0.02]; *P* < .001) and F1 score (0.27 [0.04] vs 0.34 [0.04]; *P* < .001). Of 31 variables in the model with combined variable selection, 15 (49%) were preoperative variables and 16 (51%) were intraoperative variables. The combined model demonstrated the best overall model performance, with significantly increased mean (SD) AUROC (0.79 [0.02]; *P* < .001) (Figure, A) and mean (SD) F1 (0.41 [0.04]; *P* < .001) (Figure, B) compared with the preoperative and intraoperative models.

**Table.** Variables Selected by LASSO Regularization for Inclusion in Cross-Validation Model

Preoperative		Intraoperative		Combined	
Variable^a^	Coefficient	Variable	Coefficient	Variable	Coefficient
IABP or inotropes	0.4879	IBdRBCU	0.2935	IABP or inotropes	0.4434
CHF with NYHA IV	0.1845	IBdFFPU	0.2132	IBdRBCU^b^	0.2446
Creatinine function 2	0.1611	CplegiaDeliv [antegrade]	−0.1724	CHF with NYHA IV	0.1652
Age reop function	0.1374	UER [IABP]	0.1256	IBdFFPU^b^	0.1369
Female	0.0891	IMedEACA	0.1238	Creatinine function 2	0.1166
Afib [yes]	0.0852	UER [NaN]	−0.1221	CplegiaDeliv [antegrade]^b^	−0.0965
PVD	0.0844	LwstHct	−0.1188	IMedEACA^b^	0.0906
CLD [severe]	0.0680	CplegiaDeliv [NaN]	0.0682	Shock	0.0836
Shock	0.0577	AbxSelect [exclusion]	0.0673	Age reop function	0.0749
Insufficiency [mitral]	0.0571	PrepAR [moderate]	0.0656	CplegiaDeliv [NaN]^b^	0.0707

Abbreviations: AbxSelect [exclusion], appropriate antibiotic selection [exclusion] (2280); afib [yes], atrial fibrillation [continuous/present] (980); age reop function, age at reoperation as described in Shahian et al^2^; CHF with NYHA IV, congestive heart failure and is New York Heart Association class IV; CLD [severe], chronic lung disease [severe] (405); CplegiaDeliv [antegrade], cardioplegia delivery [antegrade] (2440); CplegiaDeliv [NaN], cardioplegia delivery [NaN] (2440); creatinine function 2, creatinine function as described in Shahian et al^2^; female, sex [female] (75); IABP or inotropes, patient requires intra-aortic balloon pump or inotropes preoperatively (3725); IBdFFPU, fresh frozen plasma units (2525); IBdRBCU, red blood cell units (2520); IMedEACA, ε aminocaproic acid [yes] (2550); insufficiency [mitral], mitral valve insufficiency [moderate or severe] (1680); LwstHct, lowest intraoperative hematocrit (2315); NaN, data element missing or not recorded; PrepAR [moderate], intraoperative aortic insufficiency [moderate] (2565); PVD, peripheral vascular disease [yes] (505); shock, cardiac shock at time of procedure [yes] (930); UER [IABP], status emergent [IABP] (1990); UER [NaN], status emergent [other] (1990).

^a^ Variables are sorted by the absolute value of the associated standardized coefficient. Variable abbreviation definitions include the associated variable ID (from Society of Thoracic Surgeons Adult Cardiac Surgery Database version 2.81) provided in parentheses and the associated categorical variable provided in brackets.

^b^ Intraoperative variables.

**Figure.**
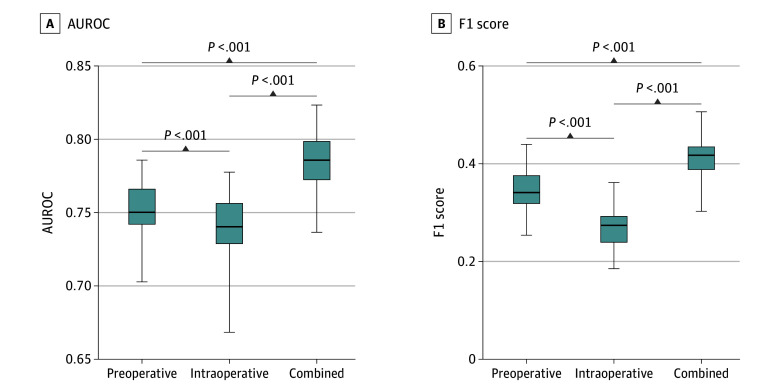
Performance of Preoperative, Intraoperative, and Combined Models AUROC indicates area under the receiver operating characteristic curve; boxes, median and interquartile range; whiskers, range.

## Discussion

This cohort study found that intraoperative data combined with preoperative data were associated with increased performance of postoperative risk stratification for a composite profile of adverse events compared with preoperative data alone. While intraoperative variables may be associated with different levels of overall quality of care provided, the use of intraoperative variables, as proposed in this study, remains limited to postoperative care guidance. In contrast, assessment of surgical quality should be limited to information available up to the time of surgical treatment. As postoperative treatment planning becomes increasingly dependent on multidisciplinary care teams, continuously updated perioperative risk models represent a novel opportunity for personalized perioperative care.^6^ Limitations of this study include the limited number of composite adverse events, the need for validation at other sites, the lack of rigorous assumption checks for intraoperative variables, and the inclusion of procedures with concomitant valve replacement, which limits generalizability. Subsequent studies may use national data repositories to generalize to larger populations, identify novel intraoperative variables, and investigate whether updated risk models in the recovery area are associated with improved postoperative outcomes.
